# Ubiquitin-Associated Protein 1 (UBAP1) Gene Mutation in a 36-Year-Old Filipino Male With Spastic Paraplegia: A Case Report

**DOI:** 10.7759/cureus.77256

**Published:** 2025-01-10

**Authors:** Kruzette Khloe L Solijon, Joshua Emmanuel E Abejero

**Affiliations:** 1 Department of Internal Medicine, Section of Neurology, Chong Hua Hospital, Cebu, PHL

**Keywords:** autosomal dominant, filipinos, hereditary spastic paraplegia, juvenile onset, ubap1

## Abstract

Hereditary spastic paraplegia (HSP) is a rare neurodegenerative disease caused by retrograde degeneration of the corticospinal tract and posterior columns, which presents with progressive bilateral leg weakness and spasticity. HSP is inherited in an autosomal dominant pattern involving over 80 causative genes. The recently identified causative gene is the ubiquitin-associated protein 1 (*UBAP1*)gene, which is associated with juvenile-onset pure spastic paraplegia-80 (SPG80). Here, we present a 36-year-old Filipino male with pure-form HSP caused by a nonsense mutation (NM 001171201.1:c.704 >G) in the *UBAP1* gene. To our knowledge, this mutation represents a new variant associated with the *UBAP1*-related HSP. This case report highlights the importance of awareness among individuals and their families affected by HSP and discusses the risks for future generations and available treatment options.

## Introduction

Hereditary spastic paraplegia (HSP) is a rare type of neurodegenerative disease characterized by retrograde degeneration of the corticospinal tract and the posterior columns [1]. This condition presents with progressive bilateral leg weakness and spasticity. The prevalence rate worldwide is 0.1 to 9.6 per 100,000 persons [2]. HSP is classified according to the signs and symptoms, age of onset, mode of inheritance, and gene involvement [3]. The signs and symptoms can either be pure or complex. The findings of progressive lower-extremity spasticity and weakness, poor vibration sense, or urinary disturbances are described in pure HSP. With complex forms, neurologic and non-neurologic features are involved. The neurologic features may include cerebellar signs, peripheral neuropathy, cognitive impairment, epilepsy, myopathy, extrapyramidal features, and brain or spinal cord defects. The reported non-neurologic features consist of ophthalmologic and orthopedic abnormalities [4,5]. The onset of symptoms may vary from early childhood to middle adulthood, with its first peak at five years old and second peak at 40 years old [6].

Approximately 75-80% of HSP cases have an autosomal dominant trait involving more than 80 causative genes [4]. The most common mutation identified is the *SPAST* gene, which encodes the protein spastin. Spastin regulates the cytoskeleton in long axons by breaking longer microtubules into shorter ones [7]. Meanwhile, the ubiquitin-associated protein 1 (*UBAP1*) gene at the 9p13.3 chromosome is a newly discovered gene mutation known to cause HSP. *UBAP1* is a component of the endosomal-sorting complex required for transport I (ESRT-I), which is responsible for the vesicular sorting of epidermal growth factors and other cellular functions [8]. The truncated mutations in *UBAP1* results in early-onset HSP, also known as juvenile-onset pure spastic paraplegia-80 (SPG80) [9]. Based on the 100,000 Genome Project, the *UBAP1* gene mutation accounts for 1.7% of 417 families with HSP [10]. Twenty variants of *UBAP1* mutations are associated with SPG80. Mostly, these are the c.425_426delAG and c.426_427delGA variants seen in families from Germany, Iran, Japan, and China [10-12].

There were no records of the prevalence of HSP in the Philippines. To date, there were only two families in Cebu province who were clinically diagnosed with familial spastic paraplegia without genetic confirmation. In both families, the symptoms appeared during the first years of life; these involved spasticity in the lower and upper extremities, resulting in difficulty ambulation. Other symptoms included foot deformities and developmental delays [13]. In addition, there were case reports of two siblings of Filipino descent in the United States who presented with early-onset spastic paraplegia, dysmorphic features, developmental delays, movement disorders, and gait abnormalities. Whole-exome sequencing of genes revealed a homozygous mutation c.364_365delAT in SPG20, confirming the diagnosis of Troyer syndrome, a complex form of HSP [14]. Currently, these cases represent all available data regarding HSP among Filipinos.

Here, we report a case of a 36-year-old Filipino male who presented with pure-form HSP caused by a nonsense mutation (NM 001171201.1:c.704 >G) in the *UBAP1* gene. To our knowledge, this is a new variant in *UBAP1*-related HSP.

## Case presentation

**Figure 1:**
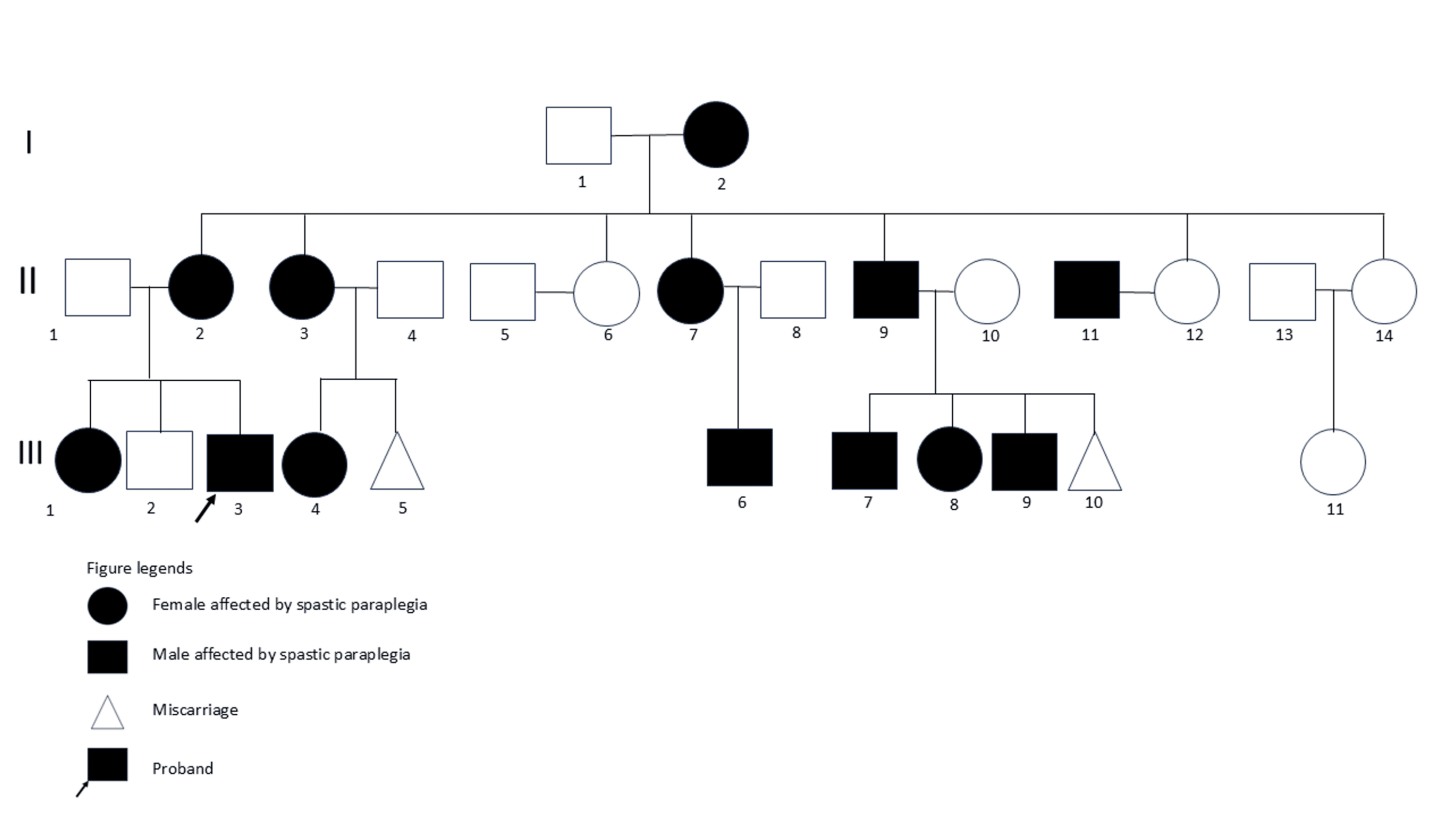
Third-degree generation pedigree of the proband (III-3) with spastic paraplegia.

The proband, identified as III-3 in Figure 1, was a 36-year-old Filipino male who sought consultation at the clinic for bilateral lower extremity weakness that started at the age of nine. Over the years, this condition gradually progressed, causing mild problems in mobility and walking. By middle 30s, his symptoms stabilized and did not worsen. Notably, he had no balance difficulties, paresthesia, muscle cramps, urinary or bowel incontinence, visual problems, and cognitive decline. Neither there was a medical history of trauma nor other illnesses like stroke and epilepsy. The physical exam was unremarkable, with no cataracts, pigmented skin lesions, dysmorphic features, and skeletal abnormalities presenting as foot deformities and kyphoscoliosis. The Montreal Cognitive Assessment (MOCA) score was 27/30, indicating no cognitive impairment. All cranial nerves were intact, revealing no loss of visual acuity, ophthalmoplegia, dysarthria, deafness, or dysphagia. The muscle bulk, tone, strength, and deep tendon reflexes were normal in the upper extremities. However, there was muscle atrophy and spasticity in the bilateral lower extremities. Muscle strength demonstrated symmetric weakness on hip flexion (4/5), hip extension (4/5), hip abduction (4/5), hip adduction (4/5), knee extension (4/5), knee flexion (4/5), ankle dorsiflexion (4/5), plantar flexion (4/5), and toe extension (4/5). Moreover, deep tendon reflexes were hyperreflexic and were graded at +3 on both knees and +4 on both ankles. Bilateral ankle clonus and extensor toe signs were present. The sensory exam was normal on three modalities. The straight leg raise test was also negative. The abdominal, cremasteric, and anal reflexes were intact, showing no signs of urinary or bowel incontinence. There was no cerebellar ataxia manifesting as gaze-evoked nystagmus, rebound phenomenon, scanning speech, and leg dystaxia.

The proband was born to non-consanguineous parents. Third-generation pedigree history (Figure 1) reported a strong familial history of spastic paraplegia on the maternal side, including his grandmother (I-2), mother (II-2), and sister (III-1). Diagnostic work-up excluded other structural, inflammatory, and metabolic causes. Brain and spine magnetic resonance imaging results suggested no optic atrophy, thinning of the corpus callosum, cerebellar atrophy, and cervical spinal cord atrophy. An electromyography and nerve conduction study (EMG/NCS) showed increased insertional activity and spontaneous potentials in the left lumbosacral paraspinal muscles (L5/S1) and absent findings of peripheral neuropathy. The complete blood count, erythrocyte sedimentation rate, and creatine kinase levels were normal. Based on clinical grounds of a positive family and unremarkable diagnostic findings, HSP was suspected. Genetic testing was pursued utilizing the whole-exome sequencing test (Centogene®, Rostock, Germany). The method targeted a panel of 443 genes associated with hereditary ataxia and spastic paraplegia. The whole-exome sequence revealed a heterozygous pathogenic variant in the *UBAP1* gene, nonsense mutation c.704T >G p.(Leu235*), confirming an autosomal dominant SPG80. Following this diagnosis, other members of the family were advised to do genetic testing and counseling. The proband (III-3), along with his mother (II-2) and his sister (III-1), were recommended to undergo regular physiotherapy and proper nutrition. Nonetheless, these measures help alleviate symptoms and slow the progression of the disease.

## Discussion

This case presented a typical slow progressive course of symptoms consistent with autosomal dominant pure HSP. Clinical diagnosis was strongly suspected when the obligatory criteria of positive family history, progressive gait disturbance, spasticity, and hyperreflexia of the lower extremities were fulfilled. Despite this clinical suspicion, a molecular diagnosis may still be warranted to rule out other neurologic disorders, such as spinocerebellar ataxia and spinal muscular atrophy, which may share similar course and symptoms with HSP [15,16].

Juvenile-onset pure spastic paraplegia-80 (SPG80) is a type of HSP characterized by symptom onset at the age of 11 and is commonly associated with *SPAST* or *KIF1A* gene mutation [17]. The proband in this case report exhibited the exact onset of symptoms but involved a different gene mutation, the *UBAP1*. As previously stated, *UBAP1* gene mutation accounts for 1.7% of 417 families with HSP, and 20 of its variants are associated with SPG80 [10,11]. The NM_001171201.1: c.704T>G identified in the proband is considered a novel variant of the *UBAP1* gene, having no records found in the Genome Aggregation Database, Exome Sequencing Project, 1000 Genome Project, and CentoMD. The clinical symptoms of the proband demonstrated a bilateral lower extremity weakness that was initially progressive but later became static as he reached his mid-thirties. At 36, he could still walk and perform daily activities independently, a similar clinical course observed in his mother and sister. 

Contrary to the typical presentation of pure HSP, the proband, his mother and sister have denied urinary bladder dysfunction and paresthesia. A comparison of clinical and genetic features of the proband, his family, and previous case reports of Filipinos with spastic paraplegia are shown in Table 1. This represents all available data regarding HSP among Filipinos. As outlined in Table 1, the novel variant found in the proband was juvenile in onset associated with a minimal disease burden, as he demonstrated less severe symptoms and good functional outcomes. By contrast, the previously reported cases started during infancy with dysmorphic features, movement disorders, and other neurologic deficits resulting in poor functional outcomes.

**Table 1:** Clinical and genetic features of the proband, his family, and previous case reports of Filipinos with spastic paraplegia

	Proband’s Family			Arquisola, A., 1988						Butler et al., 2016	
				Family A			Family B				
	III-3	III-1	II-2	Case 1	Case 2	Case 3	Case 1	Case 2	Case 3	Case 1	Case 2
Age at onset (years)	9	12	11	<1	<1	<1	1	1	1	<1	<1
Age at exam (years)	36	41	64	5	9	12	7	12	14	8	6
Biological sex	Male	Female	Female	Male	Male	Female	Male	Male	Male	Female	Female
Spasticity											
Upper extremities	-	-	-	+	+	+	-	+	+	-	-
Lower extremities	+	+	+	+	+	+	+	+	+	+	+
Muscle weakness											
Upper extremities	-	-	-	+	+	+	+	+	+	-	-
Lower extremities	+	+	+	+	+	+	+	+	+	+	+
Developmental delay	-	-	-	+	+	+	-	-	-	+	+
Intellectual disability	-	-	-	+	+	+	-	-	-	+	+
Cranial nerves	Intact	Intact	Intact	Intact	Intact	Intact	Intact	Intact	Intact	Labial and Lingual dysarthria	Labial and Lingual dysarthria
Deep tendon reflexes	++++	++++	++++	+++	++++	+++	+++	+++	+++	+++	+++
Extensor toe sign	+	+	+	+	+	+	+	+	+	-	-
Ankle clonus	+	+	+	-	-	-	+	+	+	+	+
Sensory	Intact	Intact	Intact	Intact	Intact	Intact	Intact	Intact	Intact	Intact	Intact
Gait	Spastic	Spastic	Spastic	Wide based	Wide based	Wide based	Scissored	Scissored	Scissored	in turning of the left > right ankle and inversion with gait	in turning of the left > right ankle and inversion with gait
Bladder control	Intact	Intact	Intact	Intact	Intact	Intact	Intact	Intact	Intact	Intact	Intact
Foot deformity	-	-	-	Flat foot	Pes cavus	Flat foot	Equinovarus plantar flexion	Equinovarus plantar flexion	Equinovarus plantar flexion	-	-
Dysmorphic features	-	-	-	-	-	-	-	-	-	+ Microcephaly	+ Microcephaly
Movement disorder	-	-	-	-	-	-	-	-	-	Dystonic posturing of fingers; tremors	Dystonic posturing of fingers; tremors
Gene mutation	NM_001171201.1: c.704T>G in UBAP1	Not performed	Not performed	Not performed	Not performed	Not performed	Not performed	Not performed	Not performed	c.364_365delAT in SPG20	c.364_365delAT in SPG20
Outcome	Ambulatory	Ambulatory	Ambulatory	Bedridden	Bedridden	Ambulatory	Ambulatory requiring crutches	Bedridden	Bedridden	Ambulatory	Ambulatory

The pathophysiology of *UBAP1* gene mutation causing HSP is attributed to the failure of truncated proteins to bind with ubiquitinated proteins and sort endosomes. This failure in the ESRT-I process results in misfolded and damaged protein accumulation within the cytoplasm, causing axonal degeneration in the corticospinal tract and posterior columns.

Research on HSP therapies is ongoing, primarily focusing on genetic causes and biological mechanisms. The National Institutes of Health scientists have developed a potential gene therapy for spastic paraplegia 50 (SPG50) targeting the *AP4M1* gene mutation [18]. There is no definitive treatment yet for HSP caused by *UBAP1* gene mutation. At present, the mainstay of treatment remains symptom-based and rehabilitation therapy. 

The limitation of our study was the lack of genetic testing for the proband's family in confirming the diagnosis of *UBAP1* gene spastic paraplegia due to financial constraints. The paucity of information regarding HSP in the Philippines highlighted the need for further research and genetic studies within the local population.

## Conclusions

To our knowledge, the nonsense mutation (NM 001171201.1: c.704 >G) in the *UBAP1* gene identified in the proband was a new variant of HSP. This case report not only contributed to the body of knowledge of this rare disease but also provided invaluable insights for the proband and his family, enhancing their understanding of the condition, available treatment options, and potential risks for future generations.
